# The Data Librarian’s Handbook

**DOI:** 10.5195/jmla.2019.601

**Published:** 2019-01-01

**Authors:** Kathryn Vela

**Affiliations:** Spokane Academic Library, Washington State University, Spokane, WA, kathryn.vela@wsu.edu

Academic libraries are increasingly finding themselves in a position to provide data support services to their institutions, and as a result, many librarians are moving into new roles as data librarians. In *The Data Librarian’s Handbook*, authors Robin Rice and John Southall have used their thirty years’ combined experience as data librarians to create a well-organized guide that instructs readers on key data management concepts and provides practical advice. Both new and experienced data librarians will find this book to be useful for their day-to-day work.

The book is divided into ten chapters that lead readers from introductory topics in data management to more advanced topics related to real-world practice. It opens with an introduction to the development of data services in academic libraries, an overview of research data characteristics, and the common support duties that data librarians perform.

The book transitions into providing practical advice in areas like teaching data literacy, building a data collection, and supporting a data management policy with data support services. Later chapters discuss creation and maintenance of a data repository, common challenges associated with sensitive and confidential data, and different disciplinary attitudes about data sharing. The book concludes with a discussion of how libraries can support open scholarship and open science.

Each chapter ends with a bullet-point summary of the main concepts for quick review and includes a selection of five to eight “Reflective Questions,” which prompt the reader to engage more deeply with the content by thinking critically about current practices and identifying areas for improvement. The text includes charts, maps, tables, and illustrations to drive home key points, making the book a visually interesting read. The entire book is full of references to useful resources, articles, and organizations that readers can use to further their education in data management.

*The Data Librarian’s Handbook* is an excellent resource for academic librarians from any discipline who are new to the area of data management, and it would make a suitable textbook for library science students. It would also be a useful reference for more experienced data librarians who are interested in further developing or improving their data management services and expertise.

